# Coarse Fraction Particle Matter and Exhaled Nitric Oxide in Non-Asthmatic Children

**DOI:** 10.3390/ijerph13060621

**Published:** 2016-06-22

**Authors:** Hanne Krage Carlsen, Peter Boman, Bodil Björ, Anna-Carin Olin, Bertil Forsberg

**Affiliations:** 1 Department of Public Health and Clinical Medicine, Occupational and Environmental Medicine, University of Umeå, Umeå 90187, Sweden; boman76@hotmail.com (P.B.); bodil.bjor@envmed.umu.se (B.B.); bertil.forsberg@envmed.umu.se (B.F.); 2 Section of Occupational and Environmental Medicine, Department of Public Health and Community Medicine, Institute of Medicine, Sahlgrenska Academy at University of Gothenburg, Gothenburg 40530, Sweden; anna-carin.olin@envmed.umu.se; 3 Centre of Public Health, University of Iceland, Reykjavík 101, Iceland

**Keywords:** exhaled NO, respiratory inflammation, coarse particle matter, air pollution, children

## Abstract

Coarse particle matter, PM_coarse_, is associated with increased respiratory morbidity and mortality. The aim of this study was to investigate the association between short-term changes in PM_coarse_ and sub-clininal airway inflammation in children. Healthy children aged 11 years from two northern Swedish elementary schools underwent fraction of exhaled nitrogen oxide (FENO) measurements to determine levels of airway inflammation twice weekly during the study period from 11 April–6 June 2011. Daily exposure to PM_coarse_, PM_2.5_, NO_2_, NOx, NO and O_3_ and birch pollen was estimated. Multiple linear regression was used. Personal covariates were included as fixed effects and subjects were included as a random effect. In total, 95 children participated in the study, and in all 493 FENO measurements were made. The mean level of PM_coarse_ was 16.1 μg/m^3^ (range 4.1–42.3), and that of O_3_ was 75.0 μg/m^3^ (range: 51.3–106.3). That of NO_2_ was 17.0 μg/m^3^ (range: 4.7–31.3), NOx was 82.1 μg/m^3^ (range: 13.3–165.3), and NO was 65 μg/m^3^ (range: 8.7–138.4) during the study period. In multi-pollutant models an interquartile range increase in 24 h PM_coarse_ was associated with increases in FENO by between 6.9 ppb (95% confidence interval 0.0–14) and 7.3 ppb (95% confidence interval 0.4–14.9). PM_coarse_ was associated with an increase in FENO, indicating sub-clinical airway inflammation in healthy children.

## 1. Introduction

Studded tires wear the asphalt surface and contribute to particle matter (PM), specifically the coarse fraction PM_coarse_ between 2.5–10 microns in aerodynamic diameter (PM_2.5–10_). Short-term exposure to PM attributed to mechanical wear has been associated with emergency room visits for asthma [1] and increased mortality [2]. Stronger effects of short-term exposure to coarse particles PM_2.5–10_ were found on respiratory outcomes as asthma admissions compared to other endpoints [3] and increased respiratory morbidity and mortality in relation to higher short-term PM_2.5–10_ concentrations [4]. However, the findings have been heterogeneous and stronger associations have been reported from arid regions [3] and in European studies [4].

Nitric oxide (NO) is a signalling molecule produced by epithelial cells in the airways. In airway inflammation the fraction of exhaled nitric oxide (FENO) is increased [5]. Increased levels of FENO have also been shown to predict new-onset asthma in children [6] and new-onset wheeze in adults [7]. It has also been suggested to be a predictor for asthma in children with virus-induced wheeze [8]. Taken together, these data indicate that FENO is a relevant biomarker to assess early sub-clinical inflammation, as elevated FENO levels are present before the onset of symptoms. 

In northern Sweden, the exposure to coarse particles is unusually high during April and May when streets and highways are cleared from sand and particles produced by wintertime driving with studded tires and the sanding of roads from October, resulting in lots of wear particles which are present until the roads are cleaned in late spring. This is therefore an ideal time point to better elucidate the effect of coarse particles. We were especially interested in the effect of exposure in children, as they are potentially more vulnerable. 

FENO has been used in children to assess the effects of traffic exposure in several studies; exposure to black carbon and PM_2.5_ has been associated with increases in FENO in both healthy [9,10] and asthmatic subjects [11]. In children, PM_2.5_ was associated with elevated FENO in both healthy [12] and allergic children [13]. PM_10_ from traffic as well as biomass burning has also been associated with increased FENO in healthy children [14,15] as well in a panel of urban- and suburban-dwelling children [16], and with PM_coarse_ in asthmatic children [17]; however, the study was smaller (*n* = 58) and the setting radically different. A European study found associations between background exposure to PM_coarse_ and increased risk of pneumonia in children at ages two to three [18], whereas no assocation was found with lung funtion in children in another study [19]. The role of PM_coarse_ from studded tires on airway inflammation in healthy children has not been investigated.

The aim of this study was to investigate if there was an association between airway inflammation (FENO_50_) and PM_coarse_ in a northern Swedish town in healthy children.

## 2. Materials and Methods

### 2.1. Study Population

The study population was sampled from grades 4, 5 and 6 (age 11–12) in two different schools in Umeå, Västerbotten, Sweden. The study participants were recruited from classes which had schedules compatible with the clinical research staff’s. All eligible children were invited to participate through a letter to their parents, who gave informed consent for the participation. The study protocol was approved by the regional ethical review board (reference number: 2010-345-31M).

### 2.2. Exposure

The children’s air pollution exposure at school was estimated based on data from measuring stations located within 1500 m of the schools. PM_10_ and PM_2.5_ and ozone (O_3_) were measured at the Mården continuous measuring station using a TEOM instrument (Monitor Europe 9810, Casella Measurement, Bedford, UK) for PM. Coarse particles, PM_coarse_, were defined as the fraction between PM_2.5_ from PM_10_ and calculated by subtracting PM_2.5_ from PM_10_. Nitrogen dioxide (NO_2_), nitrogen oxides (NOx), and nitrogen oxide (NO) was measured at Västra Esplanaden measuring station using a chemilluminiscence instrument (ML 9841 B, Teledyne Monitor Labs, Englewood, CO, USA). Temperature was measured at Umeå airport, approximately 5 km away from the schools, and provided as 30 minute means and recalculated into daily means when 75% of the data were available; otherwise, it was coded as missing. Pollen was measured as daily birch pollen using a Burkard trap at the roof the University Hospital. Pollen counts were recoded into categories from the daily value (0 = 0, 1–10 = 1, 11–100 = 2, 101–1000 = 3). 

### 2.3. FENO Measurements

FENO measurements were performed on each participant twice each week during spring 2011 (11 April to 6 June) if the child was present and did not have respiratory symptoms. 

Fraction of exhaled nitric oxide was measured at the flow rate 50 mL/s (FENO_50_) using Niox Mino (Aerocrine AB, Solna, Sweden). One measurement was performed at each occasion, according to the manufacturer’s instructions. The parents of the participants answered questionnaires about respiratory health, use of asthma medication, and rhinitis (allergies to furred animals and pollen). At each measuring occasion, children were asked about cold symptoms. If possible the measurements were performed at the same hour on each occasion. 

If the FENO value was less than 5 ppb (*n* = 32), *i.e.*, the lower limit of the measurement device, it was set to 2.5 ppb. As FENO is skewed to the right, log transformed values were used in the model, the estimate was then log-transformed back after the modelling.

### 2.4. Statistical Methods

Cumulative means of pollution exposure 24, 48 and 72 h prior to the FENO measurement were calculated and used in the model. Same-day temperature and pollen counts were included in the model. In the statistical analysis, MLR (multiple linear regression) was used to examine personal covariates related to FENO as a first step. If the *p*-value was <0.25 for a variable, it was included in the initial models. Then the personal covariates of interest, sex, allergy, pollen, day of week and meteorological variables, were included in the linear mixed models, together with the pollutants, to examine if the pollutants had any effect on FENO. Subjects were included as a random effect to account for personal differences, and all other variables were included as fixed effects. 

First each pollutant’s effect on FENO was examined individually in single-pollutant models. Since PM mass and nitrogen oxides (reflecting vehicle exhaust) were represented by several variables, the multi-pollutant models were constructed for each pairwise combination of PM_2.5_ and PM_coarse_ and NO, NO_2_ and NOx which were included in the multi-pollutant models with O_3_. Each pollutant’s effect on FENO was examined with exposure windows of 24, 48 and 72 h. If a covariate changed any pollutants’ effect on FENO by more than 10% or lowered Akaike’s information criteria (AIC) it was considered to be an effect modifier and was included in the model. Model selection was based on minimizing AIC.

Sensitivity analysis of sex, allergy status and background residential exposure by quartile of modeled annual mean NOx exposure at each participant’s home address was performed. 

The results are reported as change FENO (ppb) per interquartile range (IQR) of the pollutants with a 95% confidence interval (CI). All statistical analysis was performed using PASW 18 (SPSS, Chicago, IL, USA).

## 3. Results

In total, 240 children from 11 classes in the two schools were invited to participate. One hundred and four children accepted the invitation and the participation rate was 43%. Nine children had asthma or were treated with anti-inflammatory medication and were excluded from the present analysis. In total, 95 children free of asthma were included in the study group of which 46 (48%) were females, and in all, 23 (24%) had allergic rhinitis. In total, 973 FENO measurements were made on non-asthmatic children free of respiratory symptoms, five measurements per individual on average. All participants were 11 years old at the beginning of the study period. The mean FENO level in the population was 13.3 ppb (standard deviation (SD) = 10.7), higher in boys than girls (Table 1).

**Figure 1:**
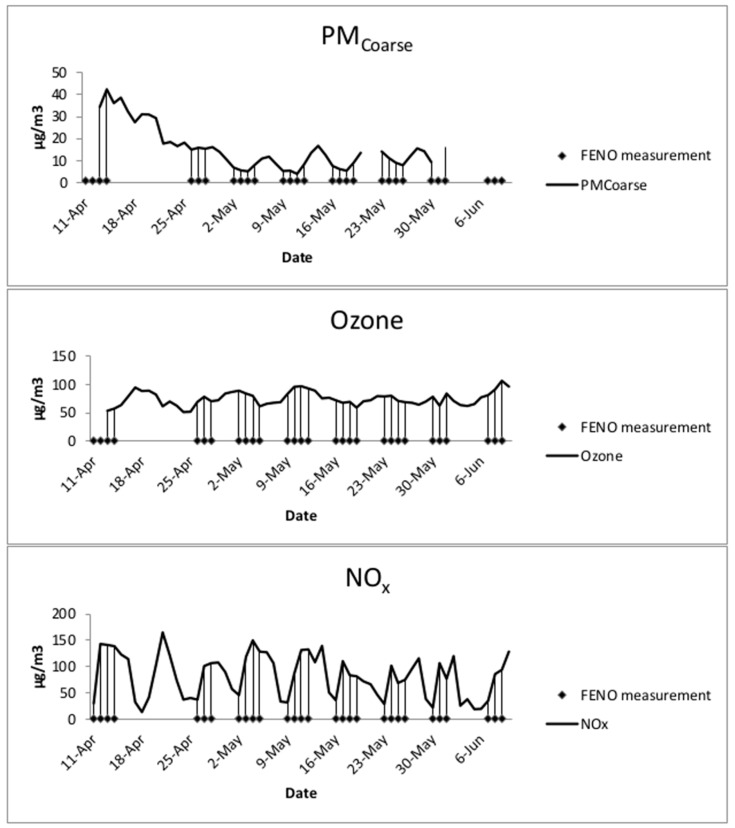
Daily concentrations of NOx, O_3_ and PM_coarse_ during the study period and indications of study days.

The study period was 62 days. PM measurements were missing the first three days and one day had missing data for NO and PM_2.5_, so these days were excluded from the analysis. The mean level of PM_coarse_ was 16.1 μg/m^3^, the mean O_3_ was 75.0 μg/m^3^, the mean NO_2_ was 17.0 μg/m^3^, the mean NO_2_ was 82.1 μg/m^3^, and the mean NO was 65 μg/m^3^ (Figure 1, Table 2). 

### 3.1. Descriptive Statistics

There were no significant correlations between 24 h means of PM_coarse_ and other pollution variables or temperature. PM_2.5_ was positively correlated with NO_2_, O_3_ and temperature. O_3_ was positively correlated with temperature. There were high correlations (>0.9) between nitrogen oxide, nitrogen dioxide and NOx (Table 3).

### 3.2. Analysis Results

Temperature increased the model fit, pollen and week day were the effect modifiers and gender was a significant predictor; these were all included in the models. Reported rhinitis did not improve the model fit and was not an effect modifier; thus it was not included in the models.

In the single-pollutant models, there were no statistically significant associations between air pollutants and FENO. Only exposure to PM_coarse_ during the previous 24 h period was near statistical significance with an estimated relative change in FENO of 6.3 ppb (95% CI: 0.5; 13.5%) per IQR change in pollutant concentration (Table 4).

In the multi-pollutant models, 24 h PM_coarse_ exposure was associated with statistically significant increases in FENO from 6.9 ppb (95% CI 0.0; 14) to 7.3 ppb (95% CI 0.4; 14.9) per IQR, depending on whether the regression was adjusted for for NO_x_, NO_2_ or NO. Seventy-two-hour NO_2_ was associated with a significantly increased FENO of 7.3 ppb (95% CI 0.6; 14.6) in the models adjusted for PM_coarse_ and O_3_. Twenty-four-hour O_3_ was, on the other hand, associated with statistically significant decreases in FENO of between −6 ppb (95% CI −12.0; −0.2) to −7.3 (95% −13.2; −1.2) in all models except in the model adjusted for PM_2.5_ and NO. The effect estimates associated with O_3_ for the previous 72 h in models adjusted for NO_2_ were −6.6 ppb and −6.7 ppb per IQR but these estimates did not reach statistical significance (Table 5). There were no significant associations between FENO and any pollutant in the 48 h pollutant models. There were no significant associations with PM_2.5_, NOx or NO in any models in any exposure window (Table 5). The daily pollen level was an effect modifier, but was not itself significantly associated with FENO and the results are not shown.

In sensitivity analyses stratified by allergy status there were no changes in the association between FENO and pollutants. The long-term residential exposure quartile of (modelled NOx) was not a significant predictor of FENO and did not modify the association with short-term exposure (data not shown).

## 4. Discussion

In this panel study of repeated FENO measurements in 95 schoolchildren over two months, we found significant within-individual increases of FENO after exposure to PM_coarse_ during the previous 24 h and NO_2_ during the previous 72 h in models adjusted for other pollutants. Exposure to O_3_, on the other hand, was associated with decreased FENO levels after adjusting for other pollutants. The results indicate that PM_coarse_, derived mainly from mechanical wear, can induce sub-clinical airway inflammation in healthy children, and it would appear that exposure 24 h before the clinical examination is most relevant. This could be due to very low background levels of PM, so even moderate exposure levels affect FENO in the study group. 

FENO is mainly associated with T-helper cell type 2 (Th2) driven airway inflammation, where an increase in airway eosinophils is a major characteristic even if the association between FENO and eosinophils is not very strong [20,21]. It seems likely that eosinophils in induced sputum and FENO reflect parallel processes in the inflamed airways. Traffic exposure, on the other hand, is merely inducing neutrophilic inflammation [22]. Nevertheless, FENO has been shown to be elevated in children with asthma living close to major roads [23], as well as after ozone exposure [24,25], and hence seems a biomarker of interest. In the current study, children with colds were excluded; nevertheless, the maximum FENO value was 71 ppb, but FENO has high variability.

PM_coarse_ is a general problem in cities in northern Sweden where sand and studded tires are used to increase driving safety on icy roads. The entire road network is an important source of PM_coarse_ in winter and spring, especially during periods of dry and windy weather where the European 24 h air quality guideline values are exceeded in the central part of Umeå, the current study setting [26]. However, regional background levels of PM are very low, which is why small absolute concentration changes present as large relative changes. Newly and locally generated particles thus become a larger fraction of PM in urban settings where most central monitors are located. Previous reports of health effects from coarse PM in urban settings could be due to variation in regional background PM (e.g., desert dust) which is reflected in concentrations measured at a single monitor rather than variation from nearby local sources, e.g., a construction site. 

A possible limitation of the current study is assigning exposure from a single, central monitor which limits the available outcome. The monitors are located near the schools, but the children’s residence may not be very close and other daytime activities may modify the children’s daily exposure. However, assigning PM_coarse_ exposure from a single, central monitor is considered to be representative for large urban areas, especially in studies of temporal variation [27]. Several studies have determined that there is a high correlation between personal exposure in children and exposure from central monitors in children and that classroom PM_10_ exposure was highly correlated with these metrics [28], and similar trends have been found for smaller particles in studies of the relationship between ambient and classroom measures [29].

For NO_2_, which increases during stagnation and cold temperatures, the cumulative 72 h exposure in models adjusted for PM_coarse_ and O_3_ yielded a higher effect estimate than at shorter lags.

Twenty-four-hour O_3_ levels were associated with reduced FENO in the multi-pollutant models. O_3_ levels correlated with levels of PM_2.5_ and temperature, but no other pollutants. O_3_ levels in this region are positively correlated with temperature. During stagnation O_3_ levels fall as nitrogen species build up. This happens especially in places where NOx levels are dominated by local sources due to a high NO/NO_2_ ratio from NO from local exhaust emissions. Higher O_3_ levels could indicate lower levels of exhaust components that are not measured such as ultrafine particles and aldehydes. This result is in contrast with results from other studies in settings with shorter exposure windows and higher O_3_ levels [24]; however, the exposure time window was different and O_3_ levels were lower in the current study. In previous studies FENO levels were positively associated with mean O_3_ levels of the previous eight hours in healthy children [24,25], and with those of the previous day in asthmatics [30]. Other epidemiological studies in adults have found associations between five-day cumulative O_3_ exposure levels and increased inflammation in the distal airways (FENO_270_), but at shorter lags, the association was not significant [31]. However, our effect estimates are similar to those reported in asthmatic children [32], where same-day and two-day average O_3_ levels were associated with significant decreases in FENO_50_. Other studies found no association between O_3_ and FENO_50_ in healthy children [12], and chamber studies of healthy adults also found no association with exposure to O_3_ [33]. A possible explanatory factor for this unexpected protective effect of O_3_ could be related to behaviour, as O_3_ levels tend to be higher during meteorological conditions with little wind, sun and relative warmth which could prompt people to spend more time outdoors and be physically active. Physical exercise was associated with lower FENO in adults even in settings with high exposure to traffic-related air pollutants [34]. The present study has a strong advantage to assess the effects as the study design, where both exposure and FENO were measured over a two-month period, allowing for effect estimates based on within-individual variation independent of variation between different schools or class rooms. 

Most previous studies were cross-sectional or cohort studies which evaluated the effects of chronic exposures. The exceptions are the studies of children in summer camps [24,25] where repeated FENO measurements were associated with eight-hour O_3_ means. Sarnat and colleagues [17] measured FENO in a panel of school children for 16 weeks and found that particle matter from traffic and other sources, rather than NO_2_, was associated with FENO. Greenwald and colleagues [35] measured FENO in a panel of elementary school students were measured weekly for 13 weeks to estimate the effects of exposure to diesel truck traffic, but no association was found in healthy children. Koenig and colleagues [36] measured children for 10 days while monitoring indoor and outdoor PM_2.5._ Steerenberg and colleagues [16] measured FENO and several other biomarkers in children in an urban school and a suburban school weekly and found associations with black smoke, PM_10_, NO, and NO_2_. 

PM_coarse_ originating from biomass has been associated with same-day increased FENO in children [15] but the effect estimates were lower than for the current study. However, in a study of same-day PM_10_, NO_2_ and black smoke [37], much higher effect estimates were found for PM_10_ than in the current study. Barraza-Villarreal and colleagues [11] reported that eight-hour exposure to PM_2.5_, NO_2_ and O_3_ was associated with increased FENO in healthy subjects by 1.16 ppb per IQR (17.5 µg/m^3^) PM_2.5_. However, the study set in Mexico City experienced much higher levels of PM_2.5_ and NO_2_ , and lower levels of O_3_. Other studies found no association between exhaled NO in healthy children and two-week NO_2_ or 48 h PM_2.5_ and elemental carbon measured at the school [38]. 

Long-term exposure to PM_coarse_ at the residence was not associated with FENO in 9–11 year-old children [39]. In a study of oxidative stress and airway inflammation in children, black carbon (BC) from combustion sources was associated with same-day measures of oxidative stress whereas 24 h and weekly exposure was associated with airway inflammation measured by FENO [9], so our observation could be due to the involvement of different mechanisms.

Finally, other factors affect FENO levels, e.g., gene-environment interactions, which have been described for the association between FENO_50_ and fine PM_2.5_ [40]. Among atopic rather than non-atopic children, associations between FENO and exposure to pollen [13] and PM_2.5_ have been found [10,11]. In our study, allergies were not a significant predictor of FENO and did not improve the model fit, and were thus discarded from the models. However, the current study setting had low levels of pollution, and the pollen levels were unusually low during the study period with a maximum 24 h mean concentration of 117 grains per m^3^.

As the few asthmatic children in the recruited population were all treated with anti-inflammatory medication that may attenuate FENO response [35], these children were excluded from the study population. Some children with rhinitis were, however, included, and we lack the information about the daily use of nasal steroids, but this seems unlikely to confound the results as we studied the effects of short-term fluctuations in air pollution.The lack of information about daily use of medication against rhinitis is not likely to be a confounding problem as we study short-term effects of fluctuations in air pollution.

Participation was dependent on parental consent, but it is unlikely that this would influence the result as all analysed children were healthy. Also, we lack information on time spent outdoors and physical exercise which could depend on weather conditions and influence both outdoor and indoor exposure, which could also affect FENO levels. 

## 5. Conclusions 

Exposure to PM_coarse_ and NO_2_ is associated with an increase in FENO in healthy children in the present study where both exposure and FENO were followed over a two-month period in a low-exposure setting. Exposure to O_3_, on the other hand, was associated with decreased FENO levels after adjusting for other pollutants. The results indicate that PM_coarse_ derived mainly from mechanical wear can induce sub-clinical airway inflammation in healthy children. The clinical significance of these results remains unclear, but is of interest for follow-up, as an increase in FENO has been associated with new-onset asthma and the role of PM_coarse_ is not well-studied.

## Figures and Tables

**Table 1:** Descriptive statistics for the participants (*n* = 95).

FENO_50_ (ppb)	N Subjects	Mean	SD	Min./Max.
All	95	13.3	10.7	3.0/71.0
Male	49	15.4	13.0	3.0/71.0
Female	46	11.2	7.3	3.0/66.0

SD: Standard deviation; ppb: Parts per billion.

**Table 2:** Exposure data (μg/m^3^) for the study period.

Exposure	N	Mean	SD	IQR	Min./Max.
PM_2.5_	60	5.6	2.6	2.6	2.3/16.7
PM_coarse_	49	16.1	9.8	9.6	4.1/42.3
NO_2_	61	17.0	7.3	12.8	4.7/31.3
NO_x_	61	82.1	41.5	77.6	13.3/165.3
NO	61	65.0	34.8	65.6	8.7/138.4
O_3_	59	75.0	12.3	17.0	51.3/106.3

SD: Standard deviation.

**Table 3:** Pearson correlation coefficients for the for the 24 h pollutant concentrations (μg/m^3^) and weather covariates.

Exposure	Ozone	PM_2.5_	PM_coarse_	NOx	NO_2_	NO	Temp.
Ozone	1						
PM_2.5_	0.415 *	1					
PM_coarse_	−0.139	0.008	1				
NO_x_	−0.041	0.257	0.150	1			
NO_2_	0.158	0.363 *	0.126	0.938 **	1		
NO	−0.083	0.232	0.153	0.997 **	0.909 **	1	
Temp.	0.423 **	0.414 *	−0.117	−0.228	−0.143	−0.242	1

* : *p* < 0.05; ** *p* < 0.01.

**Table 4:** Change in FENO (ppb) and 95% CI associated with an IQR change in pollutant concentration from a single-pollutant model adjusted for sex, temperature, pollen and day of week.

Expsoure	NOx	NO_2_	NO	PM_coarse_	PM_2.5_	O_3_
24 h average	2.8 (−1.1, 6.8)	0.1 (−3.7, 4.1)	0.3 (−0.4, 6.6)	6.3 (−0.5, 13.5)	0.5 (−1.5, 2.6)	−3.7 (−8.3, 1.2)
48 h average	2.4 (−1.6, 6.6)	1.4 (−2.3, 5.3)	3.0 (−1.7, 7.8)	−1.8 (−6.7, 3.2)	1.4 (−0.8, 3.5)	0.1 (−3.4, 3.7)
72 h average	2.0 (−0.9, 5.0)	2.0 (−1.5, 5.5)	2.4 (−1.1, 6.2)	−2.0 (−6.1, 2.3)	0.9 (−1.2, 3.1)	0.1 (−3.8, 4.1)

**Table 5:** Multi-pollutant models of combinations of pollutants (vertical strata) and exposure time windows of the change in FENO (ppb) and 95% CI associated with an IQR change in pollutant concentration adjusted for sex, temperature, pollen and day of week.

	Models					
	NOx, PM_coarse_, O_3_	NOx, PM_2.5_, O_3_	NO_2_, PM_coarse_, O_3_	NO_2_, PM_2.5_, O_3_	NO, PM_coarse_, O_3_	NO, PM_2.5_, O_3_
Exposure time	NOx	NOx	NO_2_	NO_2_	NO	NO
24 h average	3.8 (−1.1, 8.9)	2.9 (−1.9, 8.1)	1.9 (−3.7, 7.8)	1.1 (−3.9, 6.4)	3.6 (−0.6, 7.9)	2.9 (−1.3, 7.4)
48 h average	4.4 (−1.1, 9.8)	2.1 (−4.8, 9.4)	2.9 (−4.2, 11.0)	2.3 (−6.0, 11)	5.0 (−0.9, 11)	2.0 (−5.2, 9.7)
72 h average	4.2 (−0.2, 8.8)	3.1 (−1.5, 8.0)	7.3 (0.6, 14.6) *	9.1 (−0.5, 20)	4.5 (−0.6, 10.0)	2.9 (−2.0, 8.2)
	**PM_Coarse_**	**PM_2.5_**	**PM_Coarse_**	**PM_2.5_**	**PM_Coarse_**	**PM_2.5_**
24 h average	7.0 (0.1, 14.3) *	1.0 (−1.9, 4.0)	7.3 (0.4, 14.9) *	1.8 (−0.9, 4.5)	6.9 (0.0, 14) *	0.9 (−2.1, 3.9)
48 h average	−0.7 (−5.7, 4.6)	1.6 (−2.2, 5.5)	−0.7 (−5.8, 4.7)	1.8 (−1.7, 5.4)	−0.8 (−5.8, 4.5)	1.7 (−2.1, 5.6)
72 h average	−0.4 (−5.5, 5.0)	0.1 (−3.3, 3.5)	−2.2 (−6.8, 2.7)	−1.3 (−5.1, 2.7)	−0.2 (−5.4, 5.4)	0.4 (−2.8, 3.7)
	**O_3_**	**O_3_**	**O_3_**	**O_3_**	**O_3_**	**O_3_**
24 h average	−6.5 (−12.2, 0.7) *	−6.0 (−10.7, 0.3) *	−7.3 (−13.2, 1.2) *	−6.7 (−11.6, 0.7) *	−6.0 (−12.0, 0.2) *	−5.5 (−11, 0.4)
48 h average	0.6 (−4.6, 6.1)	−2.8 (−7.4, 2.0)	0.1 (−7.3, 8.0)	−3.7 (−9.8, 2.9)	1.2 (−3.7, 6.4)	−2.5 (−7.0, 2.2)
72 h average	−1.6 (−7.0, 4.1)	−2.6 (−6.6, 2.7)	−6.6 (−13.7, 1.3)	−6.7 (−14.0, 0.6)	−0.5 (−5.9, 5.1)	−2.0 (−6.9, 3.2)

* *p* < 0.05.

## Abbreviations

The following abbreviations are used in this manuscript:

FENO	Fraction of exhaled nitric oxide
NO	Nitrogen oxide
NO_2_	Nitrogen dioxide
NOx	Nitrogen oxides
O_3_	Ozone
PM	Particle matter
ppb	Parts per billion
SD	Standard deviation
Th2	T-helper cell type 2
